# Higher preoperative serum levels of PD‐L1 and B7‐H4 are associated with invasive and metastatic potential and predictable for poor response to VEGF‐targeted therapy and unfavorable prognosis of renal cell carcinoma

**DOI:** 10.1002/cam4.754

**Published:** 2016-06-12

**Authors:** Takehiko Fukuda, Takao Kamai, Akinori Masuda, Akinori Nukui, Hideyuki Abe, Kyoko Arai, Ken‐Ichiro Yoshida

**Affiliations:** ^1^ Department of UrologyDokkyo Medical UniversityTochigiJapan; ^2^ Dialysis centerDokkyo Medical University Koshigaya HospitalSaitamaJapan; ^3^ Department of UrologyNasu Red Cross HospitalTochigiJapan

**Keywords:** Axitinib, B7 family, CD28‐family receptors, immune checkpoints, receptor tyrosine kinase inhibitors, renal cell carcinoma, sunitinib

## Abstract

Renal cell carcinoma (RCC) is an immunogenic and proangiogenic cancer. Although antivascular endothelial growth factor (VEGF) therapies achieve impressive responses in some patients, many tumors eventually develop resistance to such therapy. The B7 family molecules such as CTLA‐4, PD‐1, and PD‐L1 are pivotal players in immune checkpoints that positively or negatively regulate various immune responses. Recently, immunotherapy based on blocking immune checkpoints with anti‐CTLA4, anti‐PD‐1, or anti‐PD‐L1 antibodies has been proposed as a potential new approach to the treatment of metastatic RCC. Higher expression of PD‐L1 and B7‐H4 in the tumors is associated with a poor prognosis in RCCs, however, the clinical impact of serum levels of B7 family molecules has not been elucidated in patients with metastatic RCCs receiving VEGF‐targeted agents. We assessed the preoperative serum levels of B7 family molecules, including CD80, CD86, PD‐1, PD‐L1, B7‐H3, B7‐H4, and CTLA‐4, and CD28 in RCC patients, and determined their relations with various clinicopathological characteristics. Elevated preoperative serum levels of PD‐L1 and B7‐H4 were correlated with less differentiated tumors, higher invasive and metastatic potential, a worse response to anti‐VEGF therapy, and shorter overall survival. These findings suggested that investigating preoperative serum levels of PD‐L1 and B7‐H4 might not only be useful to assess the biological aggressiveness of RCCs, but also to predict the efficacy of anti‐VEGF therapy and the eventual prognosis, indicating the future design of clinical trials of therapies targeting immune checkpoint in advanced RCCs.

## Introduction

The incidence of renal cell carcinoma (RCC) is steadily increasing and it now accounts for 2–3% of all adult malignancies 1. About 40% of RCC patients die of metastatic disease, because metastases are often present at diagnosis and relapse following nephrectomy is common 2. Although localized RCC can be cured by surgery, the prognosis is poor if distant metastases have developed. Approximately 30% of RCC patients have metastatic disease at presentation and recurrence occurs in another 30% of patients after complete resection of the primary tumor 2.

Clear cell RCC (ccRCC) is a typical immunogenic tumor. It frequently contains high levels of tumor‐infiltrating T cells and cytotoxic T cells that recognize and selectively kill tumor cells, and tumor‐specific T cells can be detected in the blood of RCC patients 3, 4, 5. Cytokines secreted by tumor cells or host cells play an important role as mediators and/or regulators of various tumor–host interactions involved in the progression of malignancy. T cells have an important role in the host immune response to malignancies, which is most evident in the case of tumors treated by immunotherapy. Cytokines are required for the proliferation and differentiation of effector T cells (Teff) and regulatory T (Treg) cells, and activation and suppression of the immune system show dynamic interaction, with various cytokines exerting effects in either direction and even doing so simultaneously. Thus, while immunocytokine therapy was the mainstay of ccRCC treatment for a long time, its effectiveness was extremely limited.

However, improvements in understanding of the molecular pathways involved have revealed that ccRCC is characterized by high constitutive production of vascular endothelial growth factor (VEGF) that induces a specific tumor vasculature. After the introduction of targeted therapy has changed the treatment available for metastatic RCC and has improved the progression‐free survival of patients by months with this disease 6. However, many tumors eventually develop resistance to targeted therapy, and only a very small minority of patients remains alive at 5 years due to secondary mutation of the target protein or compensatory changes within the target pathway that bypass the site of inhibition 7. Accordingly, there has been increasing interest in new immunotherapy modalities that diverge from this paradigm and could potentially improve the outcome of patients with metastatic RCC, including anticytotoxic T‐lymphocyte‐associated antigen 4 (CTLA4) antibody and antiprogrammed cell death 1 (PD‐1)/PD‐ligand 1 (PD‐L1) antibody 8.

Tumor cells need to evade destruction by the immune system to survive and this is now viewed as one of the “hallmarks of cancer” 9. Recent improvements in our understanding of tumor progression have shed light on new options for immunotherapy. Important insights into the limitations of T‐cell‐based anticancer immunotherapy have been provided by the discovery of inhibitory pathways termed immune checkpoints (a plethora of inhibitory pathways that are crucial for maintaining self‐tolerance and modulating the duration and amplitude of physiological immune responses), and it has been found that tumors coopt certain immune checkpoint pathways as a major mechanism of immune resistance 10. Accordingly, the blockade of such immune checkpoints may be a promising approach to the activation of antitumor immunity. The B7 and CD28 families play pivotal roles in the immune checkpoint system by activating and inhibiting costimulatory molecules that positively or negatively regulate immune responses 11, 12. Aberrant expression of coinhibitory molecules from the B7 family has been associated with reduced antitumor immunity and with immune evasion, prompting the development of therapeutic agents that can restore T‐cell function. Clinical studies investigating the effects of blocking CTLA‐4 and PD‐1/PD‐L1 have yielded exceptionally promising results in patients with advanced melanoma and other cancers, including RCC 8. However, the role of B7 family molecules in human cancers, including RCC, has been investigated by using tissue samples up to now. Since obtaining blood samples from patients is easier than harvesting tissue by biopsy, circulating biomarkers rather than tissue markers would be useful for assessing the evolution of tumor and immune responses during therapy, as well as for individualized adjustment of treatment. Therefore, we were interested in measuring the serum levels of B7 family molecules and CD28 family receptors in patients with ccRCC. We investigated the preoperative serum levels of B7/CD28 molecules simultaneously in ccRCC patients and to assess correlations with clinicopathological features and the response to treatment. Such information should shed light on the role and biological significance of immune checkpoints in human ccRCCs.

## Materials and Methods

### Patients

This is a retrospective study to investigate the preoperative serum levels of B7/CD28 molecules of the 181 patients (109 men and 72 women) in whom ccRCC was diagnosed histopathologically and nephrectomy was performed at our centers between June 2007 and June 2014. Radical nephrectomy for 118 nonmetastatic patients (cT_any_N_any_M0) or cytoreductive nephrectomy for 63 metastatic patients (cT_any_N_any_M1) were performed before any other therapy. All of them underwent preoperative imaging (CT and/or MRI) for tumor staging. The postoperative follow‐up period ranged from 3 to 100 months (median: 31 months). The tumor grade and clinical stage were determined according to the Fuhrman system and the TNM classification, respectively 13, 14. In this cohort, the study identified 63 patients with metastatic ccRCC (cT_any_N_any_M1) at diagnosed who had underwent cytoreductive nephrectomy and received tyrosine kinase inhibitors for metastatic lesions as adjuvant therapy. This study was conducted in accordance with the Helsinki Declaration and was approved by the institutional ethical review board of Dokkyo Medical University Hospital. Each patient signed a consent form that was approved by our institutional Committee on Human Rights in Research.

To be included in the study, patients were required to (1) be adults (≥18 years old) diagnosed with ccRCC; (2) be treated for 63 metastatic ccRCC at cytoreductive nephrectomy with sunitinib as first‐line therapy; (3) have discontinued the first agents for medical reasons (e.g., disease progression, nonresponse without progression, drug intolerance); (4) have subsequently initiated axitinib as second‐line therapy in; and (5) have their medical records available for review for the entire period encompassing initiation from first therapy until the most recent follow‐up or death. Postoperative adjuvant therapy with sunitinib was given to 63 patients (initial dose of 37.5 or 50 mg/day, 4 weeks on and 2 weeks off), as first‐line systemic therapy after cytoreductive nephrectomy. Within the patients refractory to first‐line sunitinib, the 40 patient received axitinib (recommended starting dose of 10 mg/day) as second‐line therapy. Progression was determined by physicians based on radiographic evidence indicating progression of tumor lesions or occurrence of new lesions, physical exams indicating worsening performance status, or cancer‐related symptoms (e.g., increased pain, fever and weight loss). Doses of sunitinib and axitinib were decreased if grade 3/4 toxicity occurred. The effect of treatment on the tumors was assessed according to RECIST criteria 15. The final date of assessment of the disease was collected from patient medical records in September 2015.

Serum samples obtained from the 181 ccRCC patients and 25 healthy controls (14 men and 11 women aged 22–73 years) were stored at −80°C until analysis. The serum levels of B7 family molecules, CD28, and VEGF were measured by using the following ELISA kits: human CD80 (CSB‐E15768h, Cusabio Biotech, Wuhan, China), human CD86 (CSB‐E08542h, Cusabio Biotech, Wuhan, China), human B7‐H4 (CSB‐E15013h, Cusabio Biotech, Wuhan, China), human PD‐1 (CSB‐E13643h, Cusabio Biotech, Wuhan, China), human PD‐L1 (CSB‐E13644h, Cusabio Biotech, Wuhan, China), human CD28 (CSB‐E09296h, Cusabio Biotech, Wuhan, China), human B7‐H3 (DB7H30, R&D Systems, Minneapolis, MN), human VEGF (DVE00, R&D Systems Minneapolis, MN), and human CTLA‐4 (KA0165, Abnova, Taipe, Taiwan), as described previously 16. The mean value for three blood samples was used.

### Statistical analysis

Baseline characteristics were compared using Wilcoxon rank‐sum tests for continuous variables and chi‐square tests for categorical variables. Regarding the relationship between preoperative serum levels and variables, statistical analysis was performed by using the Mann–Whitney *U*‐test to compare two groups and the Kruskal–Wallis test for comparisons among three or more groups. Spearman's rank correlation coefficient analysis was employed to determine the relations between levels of the molecules of interest. Cause‐specific survival curves were drawn for the ccRCC patients by the Kaplan–Meier method and differences in survival were examined by the log‐rank test. The impact of T‐cell regulatory molecules, tumor grade, pT stage, pN stage, microscopic vascular invasion, and distant metastasis (M stage) on survival was assessed by Cox proportional hazards analysis using univariate and multivariate models. In all analyses, a *P* value <0.05 was considered to indicate significance. Data were analyzed with commercially available software, as described previously 16.

## Results

### Preoperative serum levels of B7 family molecules, CD28, and VEGF

**Figure 1:**
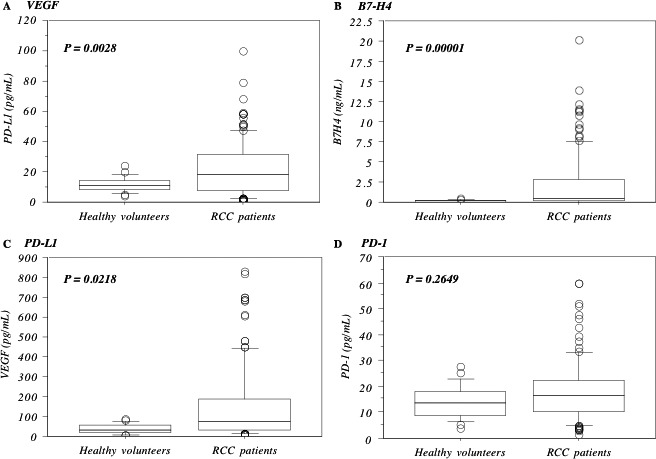
Preoperative serum levels between healthy volunteers and RCC patients. Preoperative serum levels of VEGF (A), B7‐H4 (B) and PD‐L1 (C) were higher in RCC patients than those in healthy volunteers, whereas those of PD‐1 (D) were not. The median value is the central line, the box is the interquartile range, the bars are the full range, and the points are the outliers. Bold circled *P* values were obtained by comparing the two groups with the Mann–Whitney *U*‐test.

The preoperative serum levels of B7‐H4, PD‐L1, and VEGF were significantly higher in ccRCC patients than in the healthy controls (Fig. 1 and Table 1), but those of PD‐1, B7‐H3, CTLA‐4, CD28, CD80, and CD86 were not (Fig. 1, Tables 1 and S1).

**Table 1:** Relationship between molecules and clinocopathologic features

	VEGF (pg/mL)mean ± S.D	*P* value	B7‐H4 (B7.x) (ng/mL)mean ± S.D	*P* value	PD‐1 (pg/mL)mean ± S.D	*P* value	PD‐L1 (pg/mL)mean ± S.D	*P* value
RCC patients (*n* = l81)	151.0 ± 192.9	***0.0028***	2.272 ± 3.556	***0.00001***	18.1 ± 12.2	0.2649	22.5 ± 18.5	***0.0218***
Healthy volunteers (*n* = 25)	38.1 ± 23.7		0.141 ± .121		14.1 ± 6.3		11.6 ± 5.0	
Karnofsky performance status								
>80% (*n* = 140)	89.9 ± 123.5	***0.00008***	1.402 ± 3.172	***0.00003***	17.2 ± 11.9	0.2721	17.2 ± 14.6	***0.0001***
<80% (*n* = 41)	292.4 ± 245.9		4.287 ± 3.619		20.0 ± 12.8		34.7 ± 20.9	
Grade								
1 (*n* = 18)	38.4 ± 34.9	***0.00007***	0.553 ± 0.920	***0.00003***	15.8 ± 12.2	0.1298	13.2 ± 12.2	***0.0004***
2 (*n* = 84)	89.5 ± 109.4		1.242 ± 2.659		16.1 ± 11.0		15.8 ± 13.2	
3 (*n* = 68)	261.9 ± 227.5		4.123 ± 4.254		21.8 ± 12.2		33.9 ± 19.2	
4 (*n* = 11)	596.8 ± 146.8		8.122 ± 2.524		17.3 ± 10.3		56.9 ± 16.6	
pT								
l/2 (*n* = 103)	64.5 ± 69.1	***0.00007***	0.718 ± 1.170	***0.00002***	13.5 ± 7.4	***0.0016***	14.2 ± 11.4	***0.00008***
3/4 (*n* = 78)	233.1 ± 232.6		3.741 ± 4.348		21.6 ± 13.3		30.3 ± 20.5	
pN								
0 (*n* = 168)	122.4 ± 172.6	***0.00004***	1.836 ± 3.336	***0.00009***	17.6 ± 11.9	0.7279	20.1 ± 17.1	***0.0007***
l/2 (*n* = 13)	318.0 ± 221.9		4.825 ± 3.827		18.3 ± 10.1		36.4 ± 21.0	
Microscopic vessel invasion (v)								
v (−) (*n* = 78)	54.5 ± 63.9	***0.00005***	0.675 ± 1.709	***0.00007***	13.5 ± 8.6	***0.0018***	11.7 ± 11.1	***0.00003***
v (+) (*n* = 103)	213.1 ± 222.6		3.301 ± 4.067		21.1 ± 12.4		29.2 ± 19.4	
Metastasis (M)								
M0 (*n* = 118)	105.1 ± 166.2	***0.0019***	1.492 ± 3.375	***0.0003***	16.1 ± 10.5	0.1316	17.8 ± 15.9	***0.0002***
M1 (*n* = 63)	225.2 ± 210.6		3.528 ± 3.529		20.2 ± 12.8		29.9 ± 20.2	
Adjuvant therapy								
Sunitinib (1st line): CR/PR/SD>24w* (*n* = 36)	213.3 ± 139.5	***0.0382***	1.611 ± 1.878	***0.0024***	19.7 ± 6.4	0.6531	21.1 ± 3.1	***0.0216***
Axitinib (2nd line): CR/PR/SD>24w* (*n* = 23)	221.7 ± 168.4		2.062 ± 2.590		21.5 ± 9.5		26.7 ± 18.4	
Axitinib (2nd line): SD<24w/PD* (*n* = 17)	512.4 ± 198.4		6.804 ± 3.707		20.6 ± 10.1		46.8 ± 18.2	

CR/PR/SD>24w*: complete, partial, or stable with >24 weeks response.

SD<24w/PD*: stable disease for <24 weeks or progressive disease.

The bold italic values show a statistical significance.

**Figure 2:**
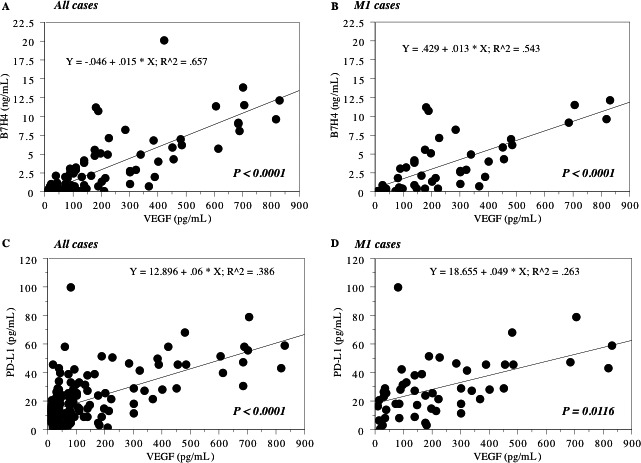
Relationship between serum B7 family molecules. Spearman rank correlation between the preoperative serum levels of VEGF and B7‐H4/PD‐L1 in all 171 cases (A and C) and in 83 metastatic (M1) cases at nephrectomy (B and D). The preoperative serum levels of B7‐H4 and PD‐1 were positively associated with those of VEGF in all case and in M1 cases.

Investigation of the correlations between preoperative serum levels of VEGF and B7 family molecules or CD28 family receptors in all cases showed that the preoperative serum VEGF level was closely associated with that of B7‐H4 (*r* ^2^ = 0.66, Fig. 2A) and weakly related with that of PD‐L1 (*r* ^2^ = 0.39, Fig. 2C), but not with the levels of PD‐1, B7‐H3, CTLA‐4, CD28, CD80, and CD86. Within metastatic (M1) cases, preoperative serum VEGF level was significantly related with that of B7‐H4 (*r* ^2^ = 0.54, Fig. 2B) and weakly related with that of PD‐L1 (*r* ^2^ = 0.26, Fig. 2D).

### Association of the preoperative serum levels of PD‐1, PD‐L1, B7‐H4, and VEGF with aggressive and metastatic RCC

The preoperative levels of PD‐1, B7‐H4, and VEGF were higher in patients with a Karnofsky performance status below 80% than in those above 80% (Table 1).

Higher preoperative serum levels of PD‐L1, B7‐H4, or VEGF were related to less differentiated tumors, local invasion, regional lymph node metastasis, microscopic vascular invasion, and distant metastasis (Table 1).

An elevated preoperative serum level of PD‐1 was correlated with local invasion and microscopic vascular invasion (Table 1).

Elevation of serum CD28 was correlated with local invasion and showed a weaker relation with microscopic vascular invasion (Table S1).

Elevation of serum CD80 and CD86 was correlated with less differentiation, local invasion, and microscopic vascular invasion (Table S1).

In contrast, serum levels of CTLA‐4 and B7‐H3 were not correlated with any of these pathological characteristics (Table S1).

### Relationship between serum levels of B7 family molecules and the response to VEGF‐targeted therapy for metastatic ccRCC

Sixty‐three patients with metastatic disease at cytoreductive nephrectomy received sunitinib as first‐line adjuvant therapy. Of 63 patients, 36 patients had clinical benefit, but 27 patients did not. Eleven patients out of latter 27 patients had best supportive care after sunitinib. Forty patients received axitinib as second‐line therapy; 23 patients showed a favorable response, whereas the other 17 patients did not. The patients with lower preoperative serum levels of PD‐L1, B7‐H4, and VEGF showed a favorable response to sunitinib or axitinib (*P *=* *0.0216, *P *=* *0.0024, and *P *=* *0.0382, respectively, Table 1).

In contrast, preoperative serum levels of PD‐1, B7‐H3, CTLA‐4, CD28, CD80, and CD86 were not associated with the response to treatment (Table S1).

### Association of PD‐L1 and B7‐H4 elevation with shorter overall survival in all ccRCC patients

**Figure 3:**
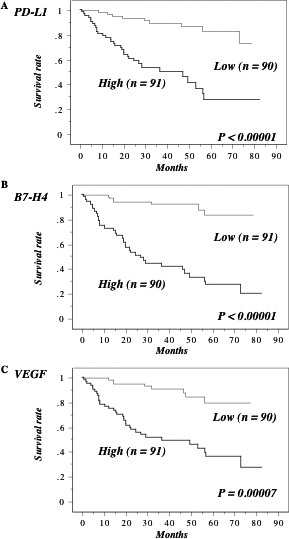
Overall survival in all 181 cases. This survival curve is based on the median values of preoperative serum levels of B7 family molecules in all 181 cases. The cases were divided into two groups at this level – high and low expression. *P* value was analyzed by log‐rank test. The patients with higher preoperative serum levels of PD‐L1 (A), B7‐H4 (B) and VEGF (C) had poorer overall survival.

The median preoperative serum level of PD‐L1 was 18.3, so the patients were divided into two groups at this cut‐off value. Kaplan–Meier analysis showed that a higher PD‐L1 level was associated with shorter overall survival (Fig. 3A). Similarly, a higher B7‐H4 level (median: 0.383) and a higher VEGF level (median: 72.6) were also associated with shorter overall survival (Fig. 3B and C). The levels of PD‐1, B7‐H3, CTLA‐4, CD28, CD80, and CD86 were not associated with overall survival. While PD‐L1, B7‐H4, VEGF, less differentiation, local invasion, regional lymph node involvement, microscopic vascular invasion, and metastasis were significant according to Cox univariate analysis, PD‐L1, B7‐H4, regional lymph node involvement, and metastasis had impact on shorter overall survival according to multivariate analysis (Table 2).

**Table 2:** Cox regression analysis for various potential prognostic factors in overall survival in all cases

Variable	Unfavorable/favorable characteristics	No. of patients	Univariate (U)			Multivariate (M)		
			Relative risk	95% confidential interval	*P* value	Relative risk	95% confidential interval	*P* value
VEGF	High/low	90/91	6.125	2.829–13.257	***0.0001***	1.023	0.214–4.896	0.9117
B7‐H4	High/low	90/91	9.637	4.275–21.725	<0.00001	3.638	1.006–13.155	***0.0489***
B7‐H3	High/low	90/91	1.838	0.930–3.629	0.2797			
PD‐1	High/low	90/91	1.689	0.864–3.305	0.1689			
PD‐L1	High/low	90/91	6.326	2.979–13.433	***<0.00001***	3.391	1.478–7.783	***0.0040***
CTLA‐4	Low/high	90/91	1.193	0.617–2.305	0.6			
CD28	High/low	90/91	2.336	0.946–4.760	0.2105			
CD80 (B7.1)	High/low	90/91	2.465	0.813–5.011	0.2297			
CD86 (B7.2)	High/low	90/91	1.378	0.705–2.696	0.3485			
Grade	4/3/2/1	11/68/84/18	4.890	2.810–8.512	***0.00001***	1.623	0.860–3.062	0.1347
pT	4,3/2,1	78/103	8.841	3.538–14.398	***0.00005***	2.193	0.613–7.840	0.2271
pN	2,1/0	13/168	7.138	2.158–24.710	<0.00001	2.164	1.023–4.580	***0.0434***
Vascular invasion	1/0	103/78	8.174	2.875–23.246	***0.00006***	1.782	1.122–5.273	0.3269
Metastasis	1/0	63/118	8.968	3.724–21.599	***<0.00001***	2.606	1.010–6.726	***0.0476***

The bold italic values show a statistical significance.

### Elevation of PD‐L1 and B7‐H4 is associated with early relapse after radical nephrectomy in nonmetastatic ccRCC patients

**Figure 4:**
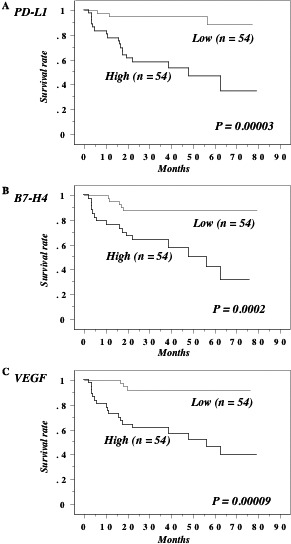
Recurrence‐free survival in 108 N0M0 cases at nephrectomy. This survival curve is based on the median values of preoperative serum levels of B7 family molecules in nonmetastatic (M0) cases at nephrectomy. The cases were divided into two groups at this level – high and low expression. *P* value was analyzed by log‐rank test. Higher preoperative serum levels of PD‐L1 (A), B7‐H4 (B) and VEGF (C) in N0M0 patients at nephrectomy were associated with shorter recurrence‐free survival.

The 108 patients with cN0M0 tumors at nephrectomy were divided into two groups based on the median preoperative serum levels of B7 family molecules, CD28, and VEGF. Comparison of the Kaplan–Meier curves for the low and high value groups revealed that high levels of PD‐L1 (median: 14.4), B7‐H4 (median: 0.318), and VEGF (median: 41.3) were associated with early relapse after radical nephrectomy (Fig. 4), whereas the other molecules did not show any association. Although less differentiation, local invasion, microscopic vascular invasion, PD‐L1, B7‐H4, and VEGF were associated with early relapse after nephrectomy according to univariate analysis, only PD‐L1 and less differentiation were significant by multivariate analysis (Table 3).

**Table 3:** Cox regression analysis for various potential prognostic factors in recurrence‐free survival in 108 N0M0 cases at radical nephrectomy

Variable	Unfavorable/favorable characteristics	No. of patients	Univariate (U)			Multivariate (M)		
			Relative risk	95% confidential interval	*P* value	Relative risk	95% confidential interval	*P* value
VEGF	High/low	54/54	8.131	2.392–27.639	***0.0008***	3.055	0.786–11.881	0.1070
B7‐H4	High/low	54/54	5.398	1.969–14.796	***0.0010***	1.680	0.532–5.301	0.3761
B7‐H3	High/low	54/54	2.105	0.502–8.827	0.3089			
PD‐1	High/low	54/54	1.936	0.232–3.776	0.9264			
PD‐L1	High/low	54/54	9.221	2.6965–31.5388	***0.0004***	5.675	1.109–29.049	***0.0372***
CTLA‐4	Low/high	54/54	1.732	0.414–7.253	0.4521			
CD28	High/low	54/54	3.625	0.730–17.997	0.1152			
CD80 (B7.1)	High/low	54/54	7.425	0.912–60.457	0.0609			
CD86 (B7.2)	High/low	54/54	1.662	0.395–6.991	0.4885			
Grade	4/3/2/1	3/23/56/266	6.041	3.011–12.123	***0.00007***	2.770	0.170–6.557	***0.0205***
pT	4,3/2,1	24/84	7.914	2.869–21.832	***0.00009***	2.414	0.485–12.0033	0.2815
Vascular invasion	1/0	42/66	6.467	2.123–19.698	***0.0010***	1.308	0.338–5.064	0.6976

The bold italic values show a statistical significance.

## Discussion

One of the most promising approaches to activating therapeutic antitumor immunity is the blockade of immune checkpoints, because it has become clear that tumors utilize these checkpoints as a mechanism of resistance, particularly against T cells specifically targeting tumor antigens 10. Recent preclinical and clinical studies have demonstrated that checkpoint blockade using anti‐CTLA‐4, anti‐PD‐1, and anti‐PD‐L1 antibodies can be successful for cancer immunotherapy 17, 18, 19. Therefore, it is important to assess whether T‐cell regulatory molecules can be used as biomarkers to predict tumor resistance and guide the choice of therapy, which is why we investigated the preoperative serum levels of B7 family molecules and CD28 family receptors in RCC patients as well as their associations with clinicopatological features.

There were four main findings of this study. First, the preoperative serum levels of PD‐L1, B7‐H4, and VEGF in ccRCC patients were significantly higher than those in the healthy controls, and the correlation of B7‐H4 or PD‐L1 with VEGF in ccRCC patients was established at their higher expression levels, in particular in metastatic disease, indicating that this correlation would have any disease‐related implications. Second, the preoperative serum levels of PD‐1, PD‐L1, B7‐H4, and VEGF were correlated with the invasive and metastatic potential of ccRCC. Third, patients with high preoperative serum levels of PD‐L1 and B7‐H4 showed a poor response to the VEGF‐targeted therapy, and had shorter overall survival according to multivariate analysis. Fourth, the higher preoperative serum level of PD‐L1 was associated with early relapse after nephrectomy according to multivariate analysis. These findings suggest that the serum levels of molecules in the B7 family might be useful for understanding the biological characteristics of ccRCC and for predicting the response to the VEGF‐targeted therapy and the survival.

Increased angiogenesis and immune system evasion are two mechanisms which help ccRCC to proliferate and metastasize. Although VEGF is primarily known for its role in promoting angiogenesis, there is mounting evidence that VEGF also has an immunosuppressive effect and that targeting tumor vasculature may increase the efficacy of immunotherapy. ccRCC can evade immune surveillance by expressing PD‐L1, which binds to PD‐1 on the surface of activated T and B cells, thus negatively regulating immune activity 20. There is a strong association between higher tissue levels of PD‐L1 expression and adverse clinical outcomes of various cancers, including RCC 11, 21, 22, 23. Dong et al. reported that PD‐L1 overexpression by tumors in vivo and in vitro leads to immune tolerance, whereas blockade of PD‐L1 or PD‐1 enhances antitumor immunity 24. Blockade of the PD‐L1/PD‐1 interaction achieves a durable response in approximately 30% of patients with ccRCC, and the response seems to be related to the tumor level of PD‐L1 expression 17, 18. However, it remains challenging to select patients who are likely to respond to anti‐PD‐1/PD‐L1 therapy 12. In the COMPARZ trial, RCC patients received sunitinib or pazopanib therapy and increased immunohistochemical tumor cell PD‐L1 expression was associated with shorter survival 25. In this study, higher preoperative serum level of PD‐L1 was correlated with poorer differentiation, invasive and metastasis profiles, and shorter overall survival, and was independent prognostic factors for overall survival. Frigola et al. reported that an elevated preoperative level of soluble PD‐L1 is associated with an increased risk of death from ccRCC, and they suggested that release of soluble PD‐L1 molecules may be a mechanism by which tumors impair systemic antitumor immunity, suggesting that inactivation or removal of soluble PD‐L1 from the serum could be clinically beneficia 26. Taken together, these findings have focused attention on blockade of PD‐1/PD‐L1 as a possible strategy for antitumor immunotherapy in patients with advanced RCC.

B7‐H4 is a negative regulatory molecule expressed on the cell membrane that inhibits proliferation and cytokine production by CD4 and CD8 T cells 11. In general, B7‐H4 positivity is associated with advanced cancer and poor survival, and this is also the case for RCC 11, 27. In an experimental model of lung metastasis, B7‐H4 expression by host cells was demonstrated to suppress tumor‐specific T‐cell responses and promote the infiltration of immunosuppressive T‐cell subsets into the lungs. As a result, B7‐H4 WT mice had more lung metastases than B7‐H4 knockout mice 28. Expression of B7‐H4 was also reported to show a correlation with tumor infiltration by Treg cells 29. Tumor‐infiltrating lymphocytes may be a manifestation of antitumor immunity, but increased tumor infiltration by T cells is associated with shorter survival of RCC patients 4, indicating that there is a potential for the mechanisms of T‐cell immunity to fail in RCC. Zhang et al. recently reported that B7‐H4 contains a functional nuclear localization sequence (NLS), which is responsible for the nuclear localization of this molecule in human RCC tissues 30. They also reported that a point mutation of the B7‐H4 NLS motif suppressed leptomycin B‐induced nuclear accumulation of B7‐H4 and abrogated B7‐H4‐mediated proliferation and cell cycle progression of HEK293 human embryonic kidney cells, whereas wild‐type B7‐H4 confers chemoresistance on RCC cell lines, indicating that nuclear localization of B7‐H4 might be crucial for its influence on cell proliferation and cell cycle progression 30. Thompson et al. reported in 101 human RCC patients that the higher serum‐soluble B7‐H4 levels were associated with poorer differentiation, local invasion, lymph node metastasis, and distant metastasis 31. Zhang et al. reported in 93 human hepatocellular carcinoma (HCC) patients that soluble B7‐H4 levels in patients were significantly higher than that in healthy controls; high sB7‐H4 levels were correlated with tumor differentiation, tumor invasion, tumor‐node metastasis stage, and poor overall survival; high sB7‐H4 levels were independent prognostic factors for overall survival 32. These findings are consistent with our observation in RCCs in this study. Thus, serum levels of B7‐H4 may be potential diagnostic and prognostic marker. Taken together, these observations provide a basis for targeting B7‐H4 in cancer and B7‐H4 is thus believed to have potential as a biomarker. Furthermore, given the Cox multivariate analyses in which PD‐L1, B7‐H4, regional lymph node involvement, and metastasis had significant impact on shorter overall survival, it might be possible to provide a better prognostic model using PD‐L1, B7‐H4 with either lymph node status or metastatic status for ccRCC patients. Similarly, since PD‐L1 and differentiation were significant for recurrence‐free survival, preoperative serum level of PD‐L1 and histological grade might be available for predicting recurrence in N0M0 cases after nephrectomy.

VEGF is a master regulator of angiogenesis, and also has profound effects on immune regulatory cell function, specifically inhibiting dendritic cells (DC) maturation and antigen presentation. For many tumors, hypoxia causes tumor cells, stromal cells, and normal cells to produce VEGF with immunosuppressive effects both locally and at distant sites, and creates a microenvironment of inhibitory inflammatory cells; the recruitment of myeloid‐derived suppressor cells (MDSC) and the induction of Treg cells by inhibiting DC maturation 33, 34, 35. On the other hand, it is not clear that PD‐L1, B7‐H4 and VEGF could evade immune surveillance in the cooperative or independent manner by negatively regulating the immune system in ccRCC, and whether VEGF is the causal factor in release of PD‐L1 or B7‐H4 expressed by RCC to peripheral blood. In this study, the preoperative serum VEGF level was associated with that of PD‐L1 and B7‐H4, and higher preoperative serum levels of PD‐L1, B7‐H4, or VEGF were related to less differentiated tumors, local invasion, regional lymph node metastasis, microscopic vascular invasion, and distant metastasis. Therefore, we hypothesize that tumors with increased VEGF expression would possess an immunosuppressive tumor microenvironment and tumor‐derived PD‐L1, B7‐H4, and VEGF may locally inactivate immune cells. However, as Frigola et al. suggested, systemic effects either by recirculation of immune cells through PD‐L1, B7‐H4, and VEGF‐positive tumor sites or by the release of biologically active soluble forms of PD‐L1, B7‐H4, and VEGF into the circulation cannot be excluded, indicating that both scenarios can contribute to global immunosuppression 26.

In contrast, the preoperative serum levels of CTLA‐4, B7‐H3, CD28, CD80, and CD86 were not correlated with tumor biological characteristics in this study. CTLA‐4 is a member of the immunoglobulin gene superfamily and is a B7‐binding protein along with its homologue (CD28) 11. It plays an important role in downregulation of T‐cell responses, T‐cell homeostasis, and maintenance of peripheral tolerance. A CTLA‐4 antibody was the first member of this class of immunotherapy agents to receive US FDA approval, and it achieved promising results in a recent phase II study of metastatic ccRCC 11. CD28 binds CD80 and CD86 expressed by activated antigen‐presenting cells and plays as the prototypic T‐cell costimulatory receptor that augments TCR‐mediated T‐cell proliferation, survival, metabolic activity, and effector function 10, 11. CD80 and CD86 are homologous costimulatory molecules predominantly expressed on antigen‐presenting cells. Interaction of these B7 family molecules with CD28 and CTLA‐4 expressed on T cells is a critical step in T‐cell activation. Despite their structural similarities, CD80 and CD86 make distinct contributions to the development of pathogenic T cells in autoimmune diseases and protective T cells in infectious diseases. While B3‐H7 shows structural similarities to the classical costimulatory molecules CD80 and CD86, there have been contradictory findings regarding its possible costimulatory or coinhibitory role. Thus, the relationship between B7‐H3 expression and the prognosis of cancer patients remains to be elucidated.

The limitations of our study are retrospective design and this study investigated a relatively small number of patients and the follow‐up period was too short to draw definite conclusions. The findings of our study need further validation in forthcoming studies, better in prospective controlled large sampled clinical trials. As biomarkers may not be static, serial measurement may be required to assess the evolution of tumor and immune responses during therapy, as well as to adjust treatment in a personalized manner. In this context, in addition to examination of the expression of PD‐L1, B7‐H4, and VEGF in the primary tumors, repeated measurement of the serum levels of B7 family molecules may be important for monitoring both tumor behavior and the condition of patients in the prospective studies on a larger scale. If we accept the hypothesis that release of soluble T‐cell suppressor molecules is a mechanism by which tumors interfere with systemic antitumor immunity, it is also important to determine whether the serum levels of soluble T‐cell regulatory molecules (such as PD‐L1, B7‐H4, PD‐1, and CTLA‐4) can be used as biomarkers to predict the effectiveness of immunotherapy targeting T cells in patients with ccRCC. Further investigation is needed to evaluate the mechanistic basis of the ability to counteract the dual function of angiogenic factors in promoting vessel growth and in suppressing immune responses by the parallel effects on host immunity and tumor vasculature in ccRCC. It is hoped that further investigation of the role of T‐cell regulatory molecules in promoting tumor immune tolerance will lead to the development of new immunotherapy options for ccRCC.

## Conflict of Interest

The authors declare that there is no conflict of interest that could be perceived as prejudicing the impartiality of the research reported.

## Supporting information


**Table S1.** Relationship between B7‐H3, CTLA‐4, CD28, CD80, and CD86 and clinocopathologic features.
